# The Median Effective Analgesic Concentration of Ropivacaine in Sciatic Nerve Block Guided by Ultrasound After Arthroscopic Anterior Cruciate Ligament Reconstruction: A Double-Blind Up-Down Concentration-Finding Study

**DOI:** 10.3389/fmed.2022.830689

**Published:** 2022-05-06

**Authors:** Cheng Xu, Fei Gu, Chengyu Wang, Yang Liu, Rui Chen, Quanhong Zhou, Jie Lu

**Affiliations:** Department of Anaesthesiology, Shanghai Jiao Tong University Affiliated Sixth People's Hospital, Shanghai, China

**Keywords:** sciatic nerve block, median effective analgesic concentration, postoperative analgesia, arthroscopic anterior cruciate ligament reconstruction, ropivacaine

## Abstract

**Background:**

The median effective analgesic concentration (MEAC; EC50 = effective concentration in 50% patients) of ropivacaine in sciatic nerve block guided by ultrasound (US) required for effective postoperative analgesia following arthroscopic anterior cruciate ligament (ACL) reconstruction has not yet been found. This study aimed to determine the effectiveness of MEAC of 20 ml ropivacaine of postoperative anesthesia for patients after ACL reconstruction.

**Methods:**

In total, 29 patients who underwent elective arthroscopic ACL reconstruction were enrolled in this study. All the subjects were given 20 ml of 0.2% ropivacaine for femoral nerve block. A concentration of 20 ml ropivacaine administered to the sciatic nerve was measured by applying the up-and-down sequential method (UDM). The starting concentration was 0.2% in the first patient, and the next patient received decremented 0.025% ropivacaine if the prior patient's postoperative visual analog pain score was <4 in the initial 8 h. Otherwise, the participant was given an incremental dose of 0.025% ropivacaine. The EC50 of ropivacaine was determined by using centered isotonic, linear-logarithmic, exponential regressions, and linear regression. The “goodness of fit” was compared among various models by calculating the residual standard errors.

**Results:**

The concentration of ropivacaine administered ranged from 0.1 to 0.2%. The EC50 [95% confidence interval (CI)] determined by four statistical methods (centered isotonic, exponential regressions, linear-logarithmic, and linear regression) was 0.115, 0.113% (0.108, 0.343%), 0.142% (0.112, 0.347%), and 0.129% (0.103, 0.359%), respectively. Among all models, the residual standard error was the smallest for the exponential regression (0.2243).

**Conclusion:**

The EC50 of ropivacaine in US-guided sciatic nerve block was 0.113–0.142%, and exponential regression model best matched the data.

## What Is Already Known About This Subject?

The previous research has investigated the application of sciatic nerve block in postoperative analgesia after anterior cruciate ligament reconstruction.

## What This Study Adds?

This research aimed to evaluate the median effective analgesic concentration (EC50 = effective concentration in 50% patients) of ropivacaine which was necessary for the successful postoperative analgesia with sciatic blockade. No similar studies have been found.

## Introduction

Anterior cruciate ligament (ACL) reconstruction may result in considerable postoperative discomfort. After ACL reconstruction, femoral nerve blockade (FNB) has been reported to provide an analgesic effect (1–3). However, many patients with FNB still complain of knee pain after ACL reconstruction. The sensory afferents of the posterior and anterior knee were provided by the articular branches of the tibial nerve and the common peroneal nerves, respectively. Thus, even in the case of an FNB, blocking common peroneal and tibial nerves through sciatic nerve block might simultaneously ameliorate pain in the anterior and posterior knees (4, 5).

The advantages of combining sciatic nerve blockade (SNB) and FNB in knee operations remain controversial (6–9). Retrospective evidence showed that compared with FNB alone, combined femoral-sciatic nerve blockade (CFSNB) before complex knee operation could enhance analgesia effects and reduce consumption of opioid (10, 11). Recent randomized controlled research on total knee arthroplasty demonstrated that CFSNB could control pain better (12, 13). Another study by Abdallah et al. (4) demonstrated that CFSNB ameliorated pain in the posterior knee following total knee arthroplasty. There are limited data, and only one randomized study has investigated the analgesic effect of sciatic block in ACL reconstruction (14).

Many clinicians are concerned about the risk of long-term neurological complications following regional anesthesia. Kew et al. (15) analyzed the effect of different nerve block techniques on lower extremity function after ACL reconstruction and found that additional SNB causes persistent knee flexor strength deficits for patients when they returned to sports after ACL reconstruction. The volume and concentration of local anesthesia might be one of the important factors. Therefore, the previous studies have been conducted to address whether it is possible to reduce the dose of local anesthesia and achieve effective nerve blocks and minimize motor blocks in the meantime (16–18).

As a long-acting local anesthetic, ropivacaine has been used for the SNB. Ropivacaine has two advantages over bupivacaine: (1) low doses and (2) similar sensory block effects as bupivacaine but less motor block and systemic toxicity (19), resulting in better conditions for knee joint functional reconstruction after ACL surgery.

This study aimed to evaluate the median effective analgesic concentration (MEAC, EC50 = effective concentration in 50% patients) of ropivacaine to achieve successful postoperative analgesia with sciatic blockade.

## Method

### Study Design and Population

This study was a prospective single-armed trial. Approval was obtained from the Ethics Committee of the Sixth People's Hospital of Shanghai [reference No. 2021-095-(1)] and registered with the Clinical Trial Registry of China (http://www.chictr.org.cn/; registration No. ChiCTR2100045439; date of registration, 15 April 2021; date of patient enrolment, 16 April 2021). All patients who underwent ACL reconstruction were assessed for eligibility. Written informed consent was collected from the eligible participants. The patients were of the American Society of Anaesthesiologists physical status I or II, and aged 18–60 years, with a body mass index of 18–30 kg/m^2^. Exclusion criteria: infection at the injection site, history of neuropathy or coagulopathy, allergic reaction to local anesthetics and opioids, dementia, history of intravenous (IV) drug abuse, preoperative chronic use of opioid, chronic pain, psychiatric diseases, those who cannot understand the scoring systems applied in this research, uncontrolled ischemic heart disease or hypertension, hepatic or renal dysfunction, and pre-existing neurologic deficits.

### Blinding Method

Experienced anaesthesiologists who had carried out not <100 FNBs guided by ultrasound (US) combined with the sciatic nerve block using a high-frequency (6–13 MHz) US probe (Sonosite, Inc., USA) performed all blocks. The procedural data were recorded by an independent investigator. The performers and investigator did not further participate in this research. An independent observer assessed the motor and sensory block effects, which were absent when the block was conducted and was blinded to the concentration of the local anesthetic injected. The same observer followed up the patients within the first 24 h after the operation.

### Methods of Block Administration

**Figure 1:**
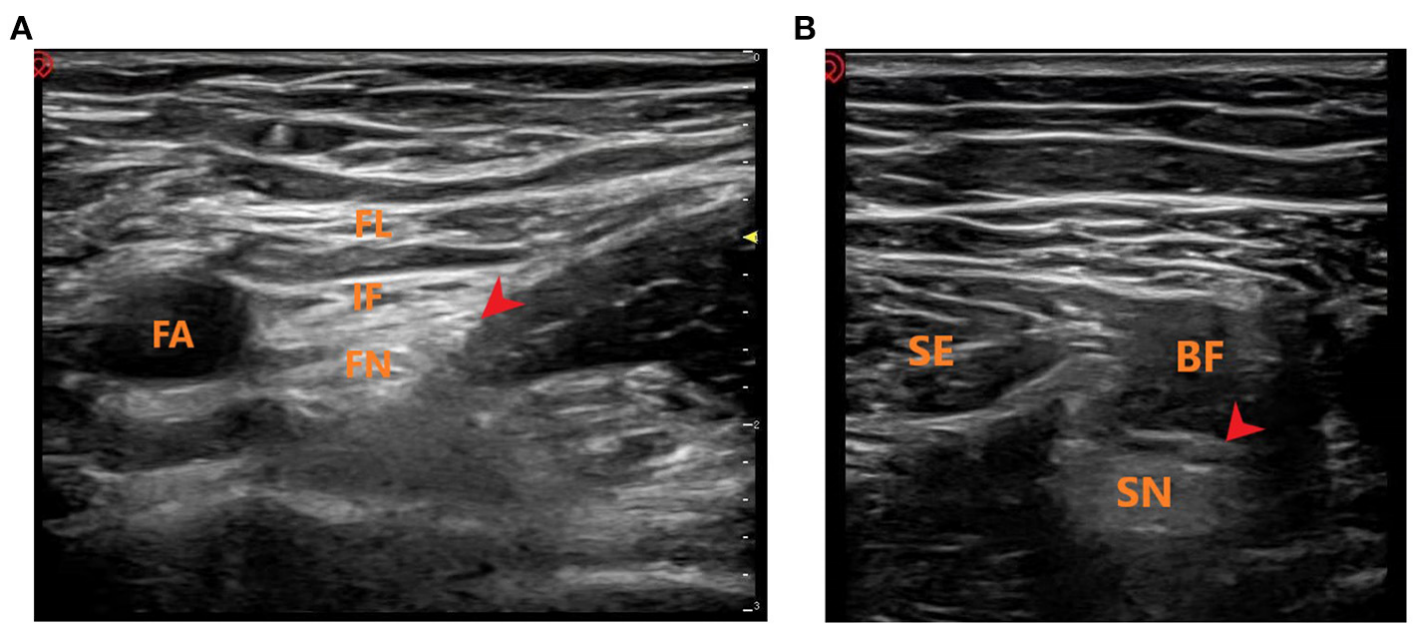
Ultrasound-guided femoral and sciatic nerve block. **(A)** Ultrasound-guided femoral nerve block. FL, fascia lataI; IF, iliac fascia; FA, femoral artery; FN, femoral nerve; Red Arrow: Local anesthetic injection site. **(B)** Ultrasound-guided sciatic nerve block. SE, semitendinosus; BF, biceps femoris; SN, sciatic nerve; Red Arrow: Local anesthetic injection site.

The peripheral nerve block was conducted under US guidance. Oxygen saturation, heart rate (HR), and blood pressure (BP) were monitored. The local anesthetic toxicity rescue kit was on the hand side. The sciatic nerve block was conducted after FNB (0.2% ropivacaine 20 ml). Patients lay in the lateral decubitus position with the operative leg on top. The injection site was in the upper-to-middle thigh or subgluteal region. The puncture site was disinfected and local anesthesia was performed as mentioned above. Around the sciatic nerve, 20 ml of ropivacaine (0.2%) was injected by using a 10-cm 21-gauge insulated needle (UniPlex NanoLine; Pajunk, Geisingen, Germany) *via* the in-plane approach, which was guided by the real-time ultrasonography (Figure 1). By applying the up-and-down small-sample sequential allocation design, the concentration of local anesthetic (20 ml ropivacaine) injected *via* the needle was measured. A concentration of 20 ml ropivacaine (0.2%) was injected into the first patient. After a successful block [in the initial 8 h after surgery, the visual analog scale (VAS) score < 4], the local anesthetic concentration decreased by 0.025% for the next patient. If the block failed, the local anesthetic concentration was increased by 0.025% for the next patient. All the participants were given ropivacaine (<3 mg/kg) in order to avoid local anesthetic toxicity.

### Clinical Procedures

The propofol (2–3 mg/kg) and sufentanil (0.1–0.15 μg/kg) were used for general anesthesia induction, and a laryngeal mask airway was placed at the proper position. Volatile anesthetic sevoflurane was used to maintain anesthesia, with the end expiratory sevoflurane concentration of above 0.7 minimum alveolar concentration (MAC) and the endtidal carbon dioxide (ETCO_2_) of 35–45 mmHg. During the operation, the anaesthesiologist would use sufentanil (0.1 μg/kg, IV) if any sign indicated insufficient anesthesia. All patients received postoperative nausea and vomiting (PONV) prophylaxis with droperidol (IV) before emergence. Patients were discharged from the hospital when the following criteria were met: (1) controlled pain with score <5, (2) patient airway with oxygen saturation >95% in room air condition, (3) diastolic and systolic BP and HR were within 20% of the levels before anesthesia, and (4) the lowest level of nausea occurred upon ingestion of clear liquids. Paracetamol (1 g) was administered every 6 h to treat analgesia after operation, and droperidol was used to prevent PONV. An independent observer blinded to the study concentration of ropivacaine recorded the VAS when the patients arrived at the ward, in the post-anesthesia care unit (PACU), and at 24 h after the surgery.

The effects of motor and sensory block in the surgical foot were assessed every 5 min post local anesthesia by an observer who was blinded to the concentration of local anesthetic injected. Successful sensory block effect was defined as no sensation to pinprick in the area with common femoral and sciatic nerve distribution and graded as follows: 1 = normal sensation (no block), 2 = blunted sensation (analgesia), and 3 = no sensation (anesthesia). Motor block was evaluated by asking the patient to dorsiflex or plantar flex their foot. The motor block effect was graded as follows: 1 = normal movement, 2 = reduced movement, and 3 = no movement (motor was blocked completely). The time from local anesthetic injection to successful block was regarded as the onset and thereby registered.

### Up-and-Down Sequential Method

A concentration of 0.2% of 20 ml ropivacaine was injected into the first patient. After the block was successful (in the initial 8 h after surgery, the VAS score was <4), the concentration of local anesthetic for the next patient was reduced by 0.025%. If the block failed (in the initial 8 h after surgery, the VAS score was no <4), the local anesthetic concentration was increased by 0.025% for the next patient. All the patients were given ropivacaine (<3 mg/kg) in order to avoid local anesthetic toxicity.

### Adverse Effect

We noted the known adverse events of using ropivacaine (arrhythmia, disturbances in hearing and vision, dizziness, dysgeusia, twitching in muscles, and modification in QRS refers to the QRS wave in the electrocardiogram) and sufentanil (urinary retention, nausea, respiratory depression, pruritus, vomiting, and sedation) in the ward and in the PACU.

### Statistical Analysis

In most cases, the exact size of samples for Dixon's up-and-down sequential method (UDM) cannot be detected in advance. When six crossovers (conversion from successful block to unsuccessful block or *vice versa*) occurred, we ceased to recruit patients (20, 21). It was observed that not <20–40 patients were asked to provide reliable estimates of the target dosage in our simulation experiments in anesthesia trials using the Dixon's UDM (22). A total of 29 patients were thus recruited in our study.

To find out the target dose ED50, three parametric estimates of the dose–responsive curve (22), linear-logarithmic, linear, and exponential regression models were used. A nonparametric model and the centered isotonic regression were also used to analyse a non-reducing dose and response relationship (23).

The residual standard error was used to find the goodness of fit, which can analyse how well the data points fit the actual model. The residual standard errors were calculated for all the models.

## Results

**Figure 2:**
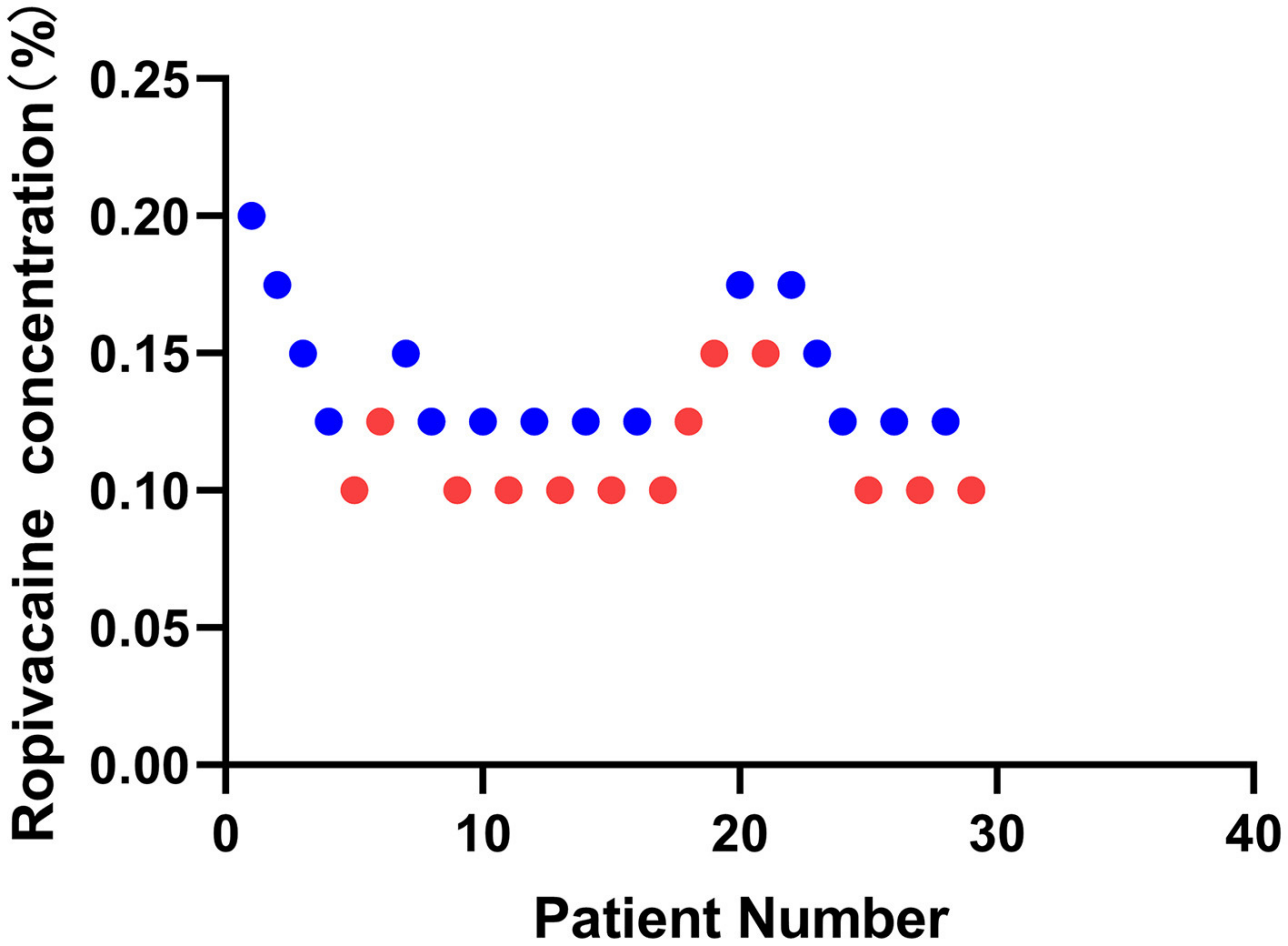
Sequential block results of ultrasound-guided sciatic nerve block using 20 ml ropivacaine according to the Dixon and Massey up-and-down method.

This study screened and included 40 patients. Thirty-two patients met the inclusion criteria, of which three patients suffered high fever on the day of the operation. Finally, a total of 29 patients were selected with 10 independent up-down deflections (Figure 2). The characteristics of the patients are demonstrated in Table 1.

**Table 1:** Patient characteristics.

**Characteristic**	**Mean ± SD or No. (%)**
Sex (male/female)	21/8
Age (year)	29.6 ± 10.75
Body mass index (kg/m^2^)	22.6 ± 1.87
ASA physical status (I/II)	15/14
Duration of surgery (min)	67.8 ± 22.02
sufentanil consumption (μg)	10.8 ± 3.33
Time to 1st rescue analgesic (h)	9.2 ± 2.71
Time to remove the laryngeal mask (min)	7.4 ± 3.27
Onset time of sensory block (min)	3.8 ± 0.93
Onset time of motor block (min)	11.4 ± 2.65
Duration of motor block (h)	8.6 ± 1.57
Analgesic satisfaction (1/2/3)	2/13/14

*ASA, American Society of Anesthesiologists*.

### Median Effective Analgesic Concentration of Local Anesthetic

**Figure 3:**
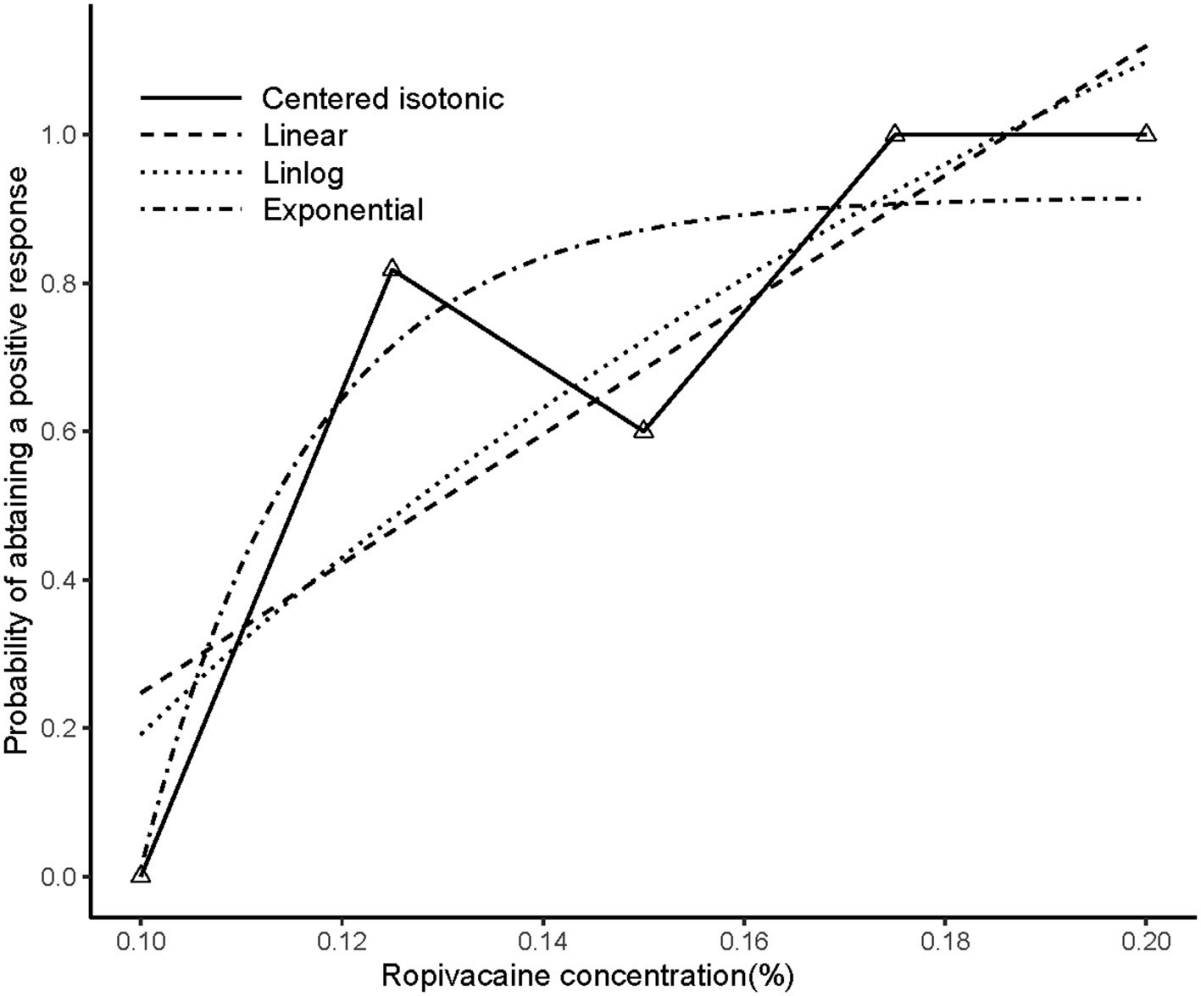
Estimated ropivacaine–sciatic nerve block relationship for a given dose level and probability of successful block. Median estimators for each model are plotted. The numbers of measurements at each ropivacaine concentration are represented by numbered triangles.

An illustration of the sequence of the failed and successful blocks is shown in Figure 3. The estimated EC50 values in linear model, linear-logarithmic model, exponential regression model, and centered isotonic regression (a nonparametric method) were 0.129, 0.142, 0.113, and 0.115%, respectively (Figure 3). The 95% CIs for the three models (exponential, linear, and linear-logarithmic models) were 0.108, 0.343%; 0.103, 0.359%; and 0.112, 0.347%, respectively (Table 2). Moreover, similar fitted probabilities were shown within the ED50 range. All the observed data were successfully covered by the 95% CIs in these models. Table 2 also showed the results of the residual standard deviations for the goodness of fit in each model. The residual standard error was the smallest (0.2243) in the exponential regression model.

**Table 2:** The median effective analgesic concentration (MEAC) and 95% confidence interval of the different models.

**Model**	**ED50 (%)**	**95% CI (%)**	**Residual standard error**
**Centered isotonic**			
Regression	0.115		
Linear	0.129	0.103, 0.359	0.2687
Linlog	0.142	0.112, 0.347	0.2443
Exponential	0.113	0.108, 0.343	0.2243

### Block Performance Characteristics

The mean onset time of the motor block and sensory block was 11.4 ± 2.65 and 3.8 ± 0.93 min, respectively. The onset time of the motor block and sensory block was not significantly different between patients with failed and successful blocks (*P* = 0.2633 and *P* = 0.1303, respectively). The average duration of the motor block was 8.6 ± 1.57 h. There was no difference in the duration of the motor block between unsuccessful and successful blocks (*P* = 0.7494).

### Postoperative Pain and Rescue Analgesia Required

**Figure 4:**
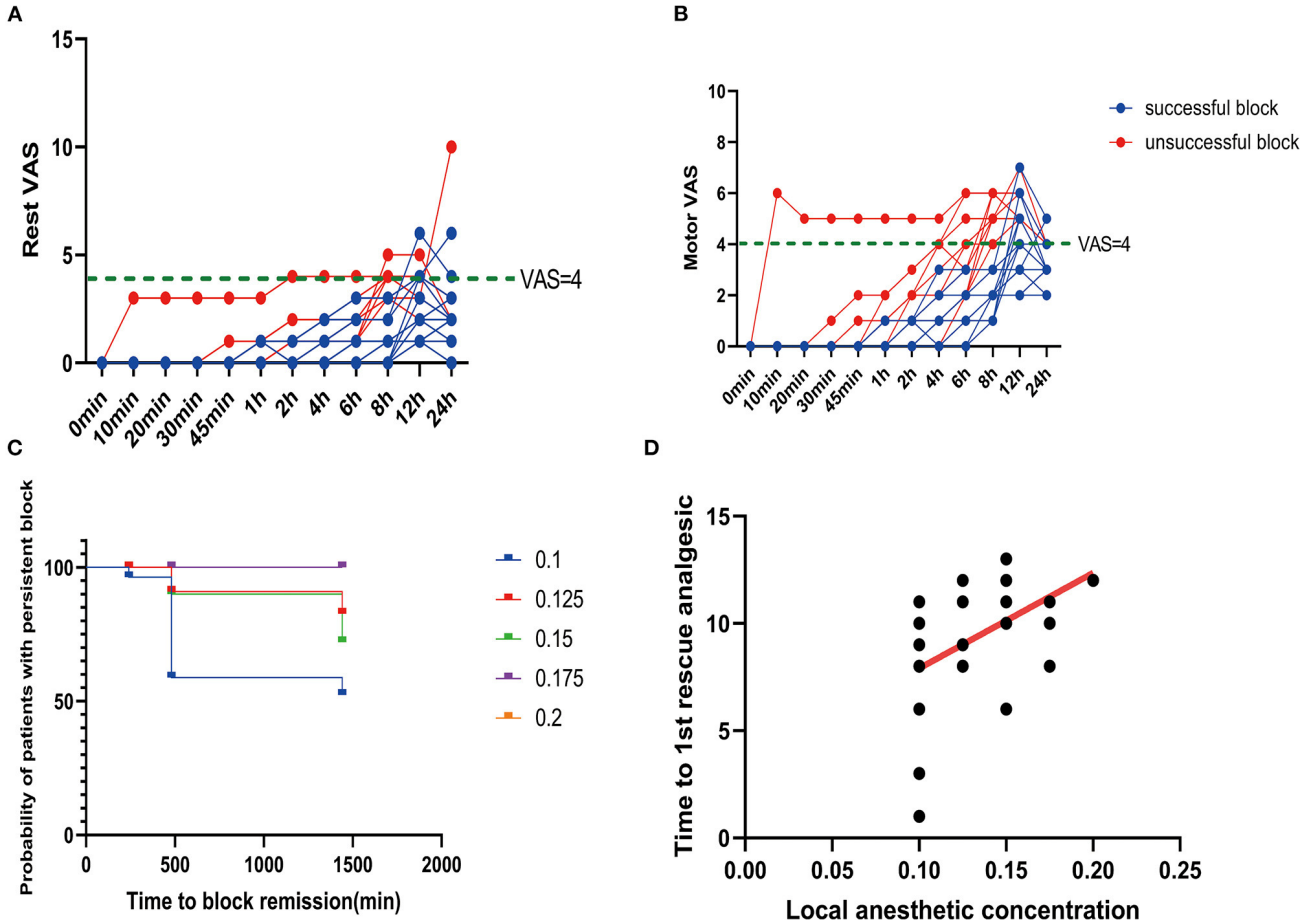
Postoperative pain scores. **(A)** Rest pain score 24 h after surgery. **(B)** Motor pain score 24 h after surgery. **(C)** Duration of the sciatic nerve block with different concentrations of ropivacaine. **(D)** Correlation between ropivacaine concentration and time to the first rescue analgesic in sciatic nerve block.

Among all the patients in this study, there were 16 successful cases of block. All the patients with successful block had a postoperative VAS score of <4 in the initial 8 h (Figures 4A,B). The average intraoperative sufentanil consumption was 10.8 ± 3.33 μg. Intraoperative sufentanil consumption was not significantly different between unsuccessful and successful blocks (*P* = 0.2579). However, the average time to the first rescue analgesic was 9.2 ± 2.71 h. The time to the first rescue analgesic was significantly different between successful and unsuccessful blocks (*P* = 0.0024). The time to the first analgesic request was moderately positively correlated with the administered local anesthetic concentration, with an r of 0.4865 (*P* = 0.0074; Figures 4C,D).

### Postoperative Adverse Events

In all patients, the femoral nerve and sciatic nerve and the spread of ropivacaine were observed, and an uncomplicated block was carried out. No adverse effect of sufentanil or ropivacaine was found. There was no PONV recorded.

## Discussion

The successful peripheral nerve block depends on the accuracy with which the nerves are impregnated and localized. However, other relevant aspects were reported to influence the rate of success and quality of nerve block, such as the volume and concentration of local anesthetic administered in proximity to the nerves (24, 25). In this research, we found that the median EC50 was 0.113% (95% CI: 0.108–0.343%).

Although the reasons of postoperative muscle weakness are multifactorial, growing evidences demonstrated that perioperative nerve blocks affect muscle strength and functional recovery after ACL reconstruction (5, 15, 26, 27). Therefore, it is necessary to explore a minimum ropivacaine concentration that can provide adequate perioperative analgesia while minimizing the degree of motor blockade. The minimum effective volume and minimum effective concentration of ropivacaine to successfully block the femoral nerve have been published (28, 29), but the minimum effective concentration of ropivacaine to block the sciatic nerve has not been identified. Eledjam's study found that the differential sensory/motor block was only evident at low concentrations (0.2% and below) (30). Based on the above information, we used a fixed FNB concentration of 0.2% in this study to investigate the EC50 of successful SNB with ropivacaine.

A variety of sciatic nerve block approaches, such as mid-femoral, subgluteal, or popliteal approaches, are conducted under the guidance of US (31, 32). Among these approaches, the subgluteal approach is advantaged. First, the depth of the puncture needle through the subgluteal approach is shallow, and US can more clearly show the course and structural characteristics of the sciatic nerve. Second, the onset time of the subgluteal sciatic nerve block is shorter than that of the popliteal sciatic nerve block, but the effect is comparable among the three groups (32).

Circumferential injections are beneficial for the sciatic nerve block guided by US (33). Compared with multiple injections of local anesthetic (LA) for circumferential spreading (34) the single-injection method was used in our study. It was found that, after injection of 20 ml of LA, all patients achieved circumferential spreading around the sciatic nerve. This is one of the reasons why the EC50 of ropivacaine concentration is low. Meanwhile, multiple injections for the circumferential spreading of the LA should be used with caution, since it may cause patient discomfort.

The previous research by Frost et al. (35) discovered that FNB could not decrease the postoperative analgesic requirements in those who had ACL reconstruction when a hamstring graft was harvested. This was anatomically meaningful because femoral block only covered the anterior knee and thigh, while hamstring graft was likely to cause pain in the posterior knee and thigh, which were the area with the sciatic sensory nerve distributed. A retrospective study demonstrated that the pain scores of patients with hamstring autografts were higher than those of patients who received allografts (11). However, Jansen et al. (14) showed no association between hamstring autografts and increased the consumption of opioid. In our research, the patients who underwent FNB combined with sciatic nerve block had low opioid consumption and PACU pain scores perioperatively. The pain in the posterior knee during ACL reconstruction might not be related to the graft harvesting site, but might be due to the operation factors (drilling a hole in the tibia, manipulation), tourniquet pain, oedema in posterior knee, or a combination of multiple factors.

A relatively high dose of local anesthetic (20 ml ropivacaine) was used in this study. Although a lower dose of local anesthetics could be applied, a high dose was used to prolong the time. There might be some adverse effects on the function of motor and early mobilization due to the dose of the local anesthetic used; however, knee mobilization should be avoided on the day of operation. Although studies have shown that the adductor canal block preserved quadriceps motor function better than FNB under an equal dose of ropivacaine. In our study, FNB with a lower concentration of ropivacaine did not affect the motor function of the quadriceps significantly.

This study had several limitations. UDM allowed the determination of an EC50 for a clinical variable with a binary outcome (36) in the small sample size study. It is well known that the UDM is unreliable when small or large percentage points are calculated, such as EC95, which is a more common indicator in clinical practice. Although the EC95 level might be more clinically useful, our simulation calculation results for 29 small samples were remarkably less accurate.

When calculating EC50 with UDM, the concentration–effect relationship is a traditional s-shaped curve, which could be incorrect. It is speculated that the EC95 is not accurate.

Thus, it was concluded that the median EC50 was 0.113%. Concentration-comparative studies are required to further investigate other volumes of ropivacaine and multiple-injection methods and to validate our findings.

## Data Availability Statement

The original contributions presented in the study are included in the article/supplementary materials, further inquiries can be directed to the corresponding authors.

## Ethics Statement

The studies involving human participants were reviewed and approved by the Ethics Committee of the Sixth People's Hospital of Shanghai [Reference No. 2021-095-(1)] and registered with the Clinical Trial Registry of China (http://www.chictr.org.cn/; Registration No. ChiCTR2100045439; date of registration, April 15, 2021; date of patient enrollment, April 16, 2021). The patients/participants provided their written informed consent to participate in this study.

## Author Contributions

CX and CW wrote the manuscript and analyzed the data. JL and QZ designed the research. CX, FG, RC, and YL performed the research. FG and RC contributed to new reagents and analytical tools. All authors agree to the submission of this manuscript.

## Conflict of Interest

The authors declare that the research was conducted in the absence of any commercial or financial relationships that could be construed as a potential conflict of interest.

## Publisher's Note

All claims expressed in this article are solely those of the authors and do not necessarily represent those of their affiliated organizations, or those of the publisher, the editors and the reviewers. Any product that may be evaluated in this article, or claim that may be made by its manufacturer, is not guaranteed or endorsed by the publisher.
